# Potent Inhibition of Zika Virus Replication by Aurintricarboxylic Acid

**DOI:** 10.3389/fmicb.2019.00718

**Published:** 2019-04-12

**Authors:** Jun-Gyu Park, Ginés Ávila-Pérez, Ferralita Madere, Thomas A. Hilimire, Aitor Nogales, Fernando Almazán, Luis Martínez-Sobrido

**Affiliations:** ^1^Department of Microbiology and Immunology, University of Rochester Medical Center, Rochester, NY, United States; ^2^Center for Animal Health Research, INIA–CISA, Madrid, Spain; ^3^Department of Molecular and Cell Biology, Centro Nacional de Biotecnología (CNB–CSIC), Campus Universidad Autónoma de Madrid, Cantoblanco, Madrid, Spain

**Keywords:** Flavivirus, Zika virus, aurintricarboxylic acid, antivirals, prophylactic, therapeutic, drug treatment

## Abstract

Zika virus (ZIKV) is one of the recently emerging vector-borne viruses in humans and is responsible for severe congenital abnormalities such as microcephaly in the Western Hemisphere. Currently, only a few vaccine candidates and therapeutic drugs are being developed for the treatment of ZIKV infections, and as of yet none are commercially available. The polyanionic aromatic compound aurintricarboxylic acid (ATA) has been shown to have a broad-spectrum antimicrobial and antiviral activity. In this study, we evaluated ATA as a potential antiviral drug against ZIKV replication. The antiviral activity of ATA against ZIKV replication *in vitro* showed median inhibitory concentrations (IC_50_) of 13.87 ± 1.09 μM and 33.33 ± 1.13 μM in Vero and A549 cells, respectively; without showing any cytotoxic effect in both cell lines (median cytotoxic concentration (CC_50_) > 1,000 μM). Moreover, ATA protected both cell types from ZIKV-induced cytopathic effect (CPE) and apoptosis in a time- and concentration-dependent manner. In addition, pre-treatment of Vero cells with ATA for up to 72 h also resulted in effective suppression of ZIKV replication with similar IC_50_. Importantly, the inhibitory effect of ATA on ZIKV infection was effective against strains of the African and Asian/American lineages, indicating that this inhibitory effect was not strain dependent. Overall, these results demonstrate that ATA has potent inhibitory activity against ZIKV replication and may be considered as a potential anti-ZIKV therapy for future clinical evaluation.

## Introduction

Zika virus (ZIKV) belongs to the genus Flavivirus within the Flaviviridae family. ZIKV is an enveloped positive sense single-stranded RNA virus with a genome size of ∼10.7 kb that encodes a single polyprotein, which is post-translationally processed by cellular and viral proteases into three structural (capsid, C; pre-membrane, prM; and envelope, E) and seven non-structural (NS1, NS2A, NS2B, NS3, NS4A, NS4B, and NS5) proteins (Tripathi et al., 2017; Avila-Perez et al., 2018).

Zika virus was initially isolated from Uganda in 1947 and viral infections only occurred sporadically in Africa and Asia until 2007. ZIKV appeared explosively as the first large-scale outbreak occurred in the Yap island in 2007 and French Polynesia in 2013 (Weaver et al., 2016). Most recently, in 2015, the first local transmission of ZIKV was found in territories of Latin America and the Caribbean, resulting in up to 1.3 million of ZIKV infection suspected cases (Tang et al., 2016; Tripathi et al., 2017).

Like other members of the Flaviviridae family, such as yellow fever virus (YFV), Dengue virus (DENV), Japanese encephalitis virus (JEV), and West Nile virus (WNV), ZIKV is commonly transmitted by the bite of infected *Aedes* mosquitos, but it can also be transmitted vertically from mother to child, through sexual contact, and in rare cases from blood transfusions (Lessler et al., 2016; Fink et al., 2018). Upon infection, ZIKV can be shed in blood, urine, semen, saliva, amniotic fluid, breast milk, and cerebrospinal fluid (Nayak et al., 2016; Colt et al., 2017; Nazerai et al., 2019). Most people (75∼80%) infected with ZIKV are asymptomatic or have mild symptoms such as fever, rash, joint pain, and conjunctivitis that can last for several days to a week (Fink et al., 2018). In rare cases, people with symptoms may have neurological Guillain-Barré syndrome complications (Oehler et al., 2014; Rivera-Concepcion et al., 2018; Nazerai et al., 2019). In the case of pregnant women, ZIKV infection can lead to microcephaly and other fetal complications as occurred during the large-scale ZIKV outbreak in Brazil in 2015 (Lessler et al., 2016). Because of the significant outbreaks in South, Central, and North America, ZIKV was declared a Public Health concern by the World Health Organization (WHO) in February 2016 (Lazear and Diamond, 2016; Ramos da Silva and Gao, 2016; Weaver et al., 2016; Tripathi et al., 2017).

There are several vaccines and antiviral drugs currently under development for the prevention or treatment of ZIKV infection (Abbink et al., 2016; Larocca et al., 2016; Shan et al., 2017; Fink et al., 2018). DNA-based (Abbink et al., 2016; Larocca et al., 2016), inactivated (Abbink et al., 2016; Larocca et al., 2016; Shan et al., 2017), live-attenuated and mRNA (Richner et al., 2017) vaccines have been proposed for the prophylactic treatment of ZIKV infections. On the other hand, arbidol (ARB) (Fink et al., 2018; Haviernik et al., 2018), bortezomib, mycophenolic acid, daptomycin (Barrows et al., 2016), obatoclax, saliphenylhalamide, gemcitabine (Kuivanen et al., 2017), emetine (Yang et al., 2018), and sofosbuvir (Bullard-Feibelman et al., 2017) have been proposed for the therapeutic treatment of ZIKV infection. Despite these tremendous efforts, there is currently no Food and Drug Administration (FDA)-approved vaccines and/or anti-viral drugs available for the treatment of ZIKV infection. Since vaccination takes at least 2 weeks to several months to show protective effects against ZIKV infection, vaccination is probably not the most appropriate prophylactic method for those who are traveling to areas where ZIKV is epidemic, endemic, or have already been infected. Moreover, vaccination may cause an important issue, such as antibody-dependent enhancement (ADE) (Bardina et al., 2017; Priyamvada et al., 2017). ADE, which has been extensively described in DENV (Priyamvada et al., 2017), is a phenomenon where preexisting antibodies facilitate binding and infection during subsequent exposure to infectious viruses, instead of neutralizing them, resulting in exacerbation of clinical signs (Bardina et al., 2017; Priyamvada et al., 2017). Because of the structural similarities between DENV and ZIKV, DENV immunity–linked ADE of ZIKV infection has also been reported (Bardina et al., 2017; Priyamvada et al., 2017). Since vaccination for ZIKV could lead to DENV ADE, antivirals could represent a better choice for the control of ZIKV infection.

Aurintricarboxylic acid (ATA), a polyanionic aromatic compound, has been shown to have inhibitory properties against several bacteria and viruses including, among others, *Yersinia pestis* (Liang et al., 2003), *Cryptosporidium parvum* (Klein et al., 2008), human immunodeficient virus (HIV) (Mitra et al., 1996; De Clercq, 2005), hepatitis C virus (HCV) (Chen et al., 2009; Mukherjee et al., 2012; Shadrick et al., 2013), Vaccinia virus (Myskiw et al., 2007), influenza virus (Hung et al., 2009), *Enterovirus 71* (Hung et al., 2010) and severe acute respiratory syndrome coronaviruses (SARS-CoV) (He et al., 2004). Mechanistic studies have suggested that ATA has the ability to modulate various cellular enzymes such as activators of the Janus kinase 2 (JAK2) and signal transducer and activator of transcription 5 (STAT5) families (Rui et al., 1998), inhibitors of nucleases (Shadrick et al., 2013), glucose-6-phosphate dehydrogenase (Bina-Stein and Tritton, 1976), and topoisomerase II proteins (Catchpoole and Stewart, 1994; Benchokroun et al., 1995) as well as the enzymatic activity of the Vaccinia virus AH1L phosphatase (Smee et al., 2010). However, to date, the ability of ATA to inhibit ZIKV infection has not been evaluated. Herein, we investigated ATA as a plausible prophylactic and therapeutic candidate against ZIKV infection. Our results demonstrate that ATA has a potent and effective antiviral activity against ZIKV in pre- and post-infection settings, including broadly antiviral activity against strains of the African and American/Asian lineages with no toxicity up to 1,000 μM in cultured cells. These data support the feasibility of implementing ATA for the treatment of ZIKV infection.

## Materials and Methods

### Cell Lines and Viruses

African green monkey kidney epithelial Vero (ATCC CCL-81) and human adenocarcinoma alveolar basal epithelial A549 (ATCC CCL-185) cells were maintained in Dulbecco’s modified Eagle’s medium (DMEM; Mediatech, Inc.) supplemented with 5% fetal bovine serum (FBS) and 1% PSG (100 U/ml penicillin, 100 μg/ml streptomycin, and 2 mM L-glutamine) at 37°C in a 5% CO_2_ atmosphere.

Paraiba/2015 ZIKV isolate was kindly provided by Stephen Dewhurst (Department of Microbiology and Immunology, University of Rochester). Uganda/1947 (MR_766 strain, Catalog No. NR-50065) and Nigeria/1968 (IbH 30656 strain, Catalog No. NR-50066) ZIKV isolates were obtained from the Biodefense and Emerging Infections Research Resources Repository (BEI Resources). Puerto Rico/2015 (PRVABC59 strain) and French Polynesia/2013 ZIKV isolates were kindly provided from the Centers for Disease Control and Prevention (CDC). Virus stocks were propagated in Vero cells and titrated by plaque assay as previously described (Marquez-Jurado et al., 2018).

### Compounds

Aurintricarboxylic acid (Catalog No. A1895) and Arbidol (ARB, Catalog No. SLM0860) were purchased from Sigma-Aldrich, MO, United States. Both compounds were prepared at 100 mM stock solution dissolved in dimethyl sulfoxide (DMSO) and kept at -20°C until experimental use. Each drug was diluted into infectious media (DMEM 2% FBS, 1% PSG) for the described experiments, where the maximum DMSO concentration was 0.1%.

### Cell Viability Assay

Cell viability in Vero and A549 cells was measured using the CellTiter 96 Non-Radioactive Cell Proliferation assay (Promega) following the manufacturer’s instructions. Briefly, confluent Vero or A549 cells (96-well plate format, 5 × 10^4^ cells/well, triplicates) were treated with 100 μl of DMEM containing serially diluted (twofold dilutions, starting concentration of 1,000 μM) chemicals or 0.1% DMSO (vehicle control). Plates were incubated at 37°C in a 5% CO_2_ atmosphere for 36 or 72 h. Samples were treated with 15 μl of Dye Solution and incubated at 37°C in a 5% CO_2_ atmosphere for 4 h. Next, cells were treated with 100 μl of Solubilization Solution/Stop Mix and absorbance at 570 nm was measured using a Vmax kinetic microplate reader (Molecular Devices, Waltham, MA, United States). Viability of compound-treated cells was calculated as a percentage relative to values obtained with DMSO-treated cells. Non-linear regression curves and the median cytotoxic concentration (CC_50_) were calculated using GraphPad Prism software version 8.0.

### Microplaque Reduction Assay and Immunostaining

Confluent monolayers (96-plate format, 5 × 10^4^ cells/well, triplicates) of Vero cells were infected with 25 plaque forming units (PFU)/well of Paraiba/2015, Uganda/1947, Nigeria/1968, Puerto Rico/2015, and French Polynesia/2013 ZIKV strains at 37°C in infection media. After 1 h of adsorption, virus inoculum was removed and cells were washed three times with infection media before adding fresh infection media containing 1% microcrystalline cellulose (Avicel, Sigma-Aldrich) and the indicated concentration of compounds, or 0.1% DMSO as vehicle control. In case of pre-treatment experiments, the cell monolayers were treated with the indicated concentration of compound, or 0.1% DMSO, for the indicated times before ZIKV infection. Infected cells were incubated at 37°C for 36–60 h, depending on virus strains. For immunostaining, cells were fixed with 4% paraformaldehyde for 1 h, washed three times with phosphate buffered saline (PBS) and permeabilized with 0.2% Triton X-100 for 10 min at room temperature. Then, the plates were blocked with 1.25% bovine serum albumin (BSA) in PBS (blocking solution) for 1 h at room temperature, followed by incubation with 1 μg/ml of the pan-flavivirus envelop (E) protein monoclonal antibody 4G2 (ATCC, Catalog No. VR-1852) diluted in blocking solution for 1 h at 37°C. After incubation with the primary antibody, cells were washed three times with PBS and developed with the Vectastain ABC kit and the DAB Peroxidase Substrate kit (Vector Laboratory, Inc., CA, United States) according to the manufacturers’ instructions. Stained plaques were analyzed using the CTL ImmunoSpot plate reader and counting software (Cellular Technology Limited, Cleveland, OH, United States). Virus titers were calculated as PFU/ml (Nogales et al., 2015). Non-linear regression curves and the median inhibitory concentration (IC_50_) were determined as described above.

### Virus Growth Kinetics

Confluent monolayers (24-well plate format, 2.5 × 10^5^ cells/well, triplicates) of Vero or A549 cells were infected (multiplicity of infection, MOI, 0.1) with Paraiba/2015 diluted in infection media for 1 h at room temperature. After viral absorption, cells were incubated with infection media containing the indicated concentrations (250, 25, 2.5, and 0 μM) of ATA. At 12, 24, 48, and 72 h post-infection (h p.i.), tissue culture supernatants were collected and titrated on Vero cells by immunostaining as described previously (Marquez-Jurado et al., 2018).

### Apoptosis Assay

Levels of apoptosis were measured using the Caspase-Glo^®^ 3/7 Assay (Promega, WI, United States) following the manufacturer’s instruction. Briefly, Vero and A549 cells (24-well plate format, 2.5 × 10^5^ cells/well, triplicates) were infected with ZIKV Paraiba/2015 (MOI of 0.1) and, at the indicated times post-infection, cells and tissue culture supernatants were collected and centrifuged. Twenty five microliters of supernatants were mixed with 25 μl of Caspase-3/7 reagent using a plate shaker, incubated at room temperature for 1 h, and luminescence at 570 nm was measured using a SpectraMax iD5 (Molecular Devices, Waltham, MA, United States) following the manufacturer’s instructions.

### Statistical Analysis

Two-way ANOVA was used to evaluate significant differences. Data are expressed as the mean ± standard deviation (SD) of at least three independent experiments in triplicates using Microsoft Excel software. Value were considered statistically significant when ^∗^*p* < 0.0332, ^∗∗^*p* < 0.0021, ^∗∗∗^*p* < 0.0002, ^∗∗∗∗^*p* < 0.0001. All data were analyzed with Prism software version 8.00 (GraphPad Software, CA, United States). CC_50_ and IC_50_ were determined using sigmoidal dose response curves (GraphPad Software, CA, United States). The selective index (SI) of each compound was calculated by dividing the CC_50_ with the IC_50_.

## Results

### Analysis of ATA Toxicity in Vero and A549 Cells

**FIGURE 1:**
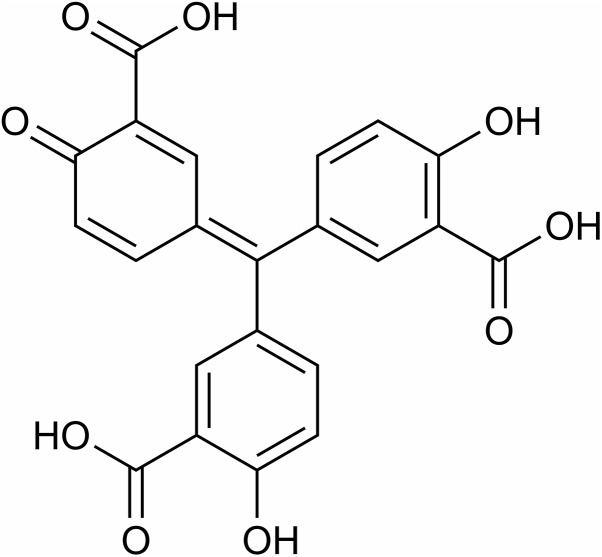
Aurintricarboxylic acid (ATA) structure. Molecular weight = 441.329 g/mol. Compound ID in PubChem: 2259.

**FIGURE 2:**
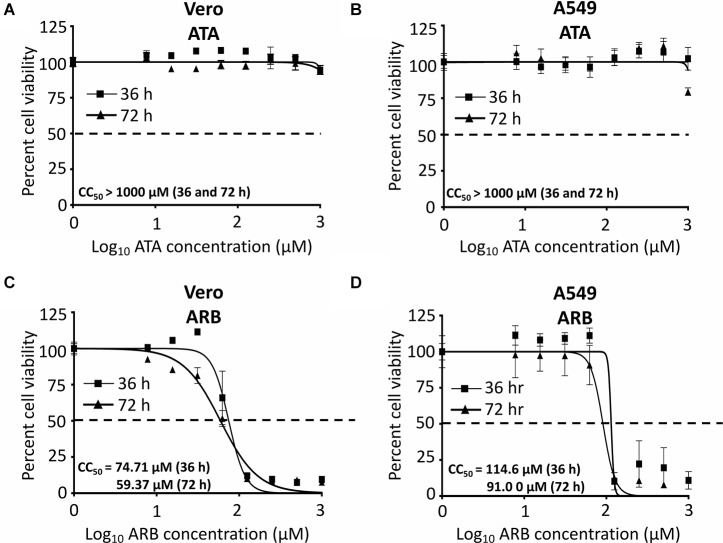
Cytotoxicity of ATA: Vero **(A,C)** or A549 **(B,D)** cells (96-well plate format, 5 × 10^4^ cells/well, triplicates) were treated with the indicated doses (twofolds dilutions, starting concentration 1,000 μM) of ATA **(A,B)** or ARB **(C,D)**. Cell proliferation assays were performed at 36 h (triangle) and 72 h (square) post-treatment and the CC_50_ for each compound was calculated at both 36 and 72 h after treatment. Dotted line indicates the 50% toxicity. Data was expressed as mean and SD from three independent experiments conducted in triplicates.

Before examining the inhibitory effect of ATA (Figure 1) against ZIKV infection, we first determined the CC_50_ of ATA on Vero and A549 cells (Figure 2). For this, we treated both cell lines with serial (twofold) dilutions of ATA and measured cell viability at 36 and 72 h post-treatment. As an internal control for these studies, we used ARB, a drug that has been previously described to have antiviral activity against ZIKV in Vero (Haviernik et al., 2018) and A549 (Fink et al., 2018) cells. We did not observe any toxicity with ATA in Vero (Figure 2A) or A549 (Figure 2B) cells at 36 or 72 h post-treatment, even at the highest concentration tested (1,000 μM), while ARB showed CC_50_ values of 74.71 ± 1.09 or 59.37 ± 1.10 μM in Vero (Figure 2C) and 114.6 ± 1.08 or 91.0 ± 1.08 μM in A549 (Figure 2D) cells (Table 1) at 36 or 72 h post-treatment, respectively.

**Table 1:** Inhibition of ZIKV replication with ATA.

ZIKV strain	Drug	Treatment	Hours	Cells	CC_50_ (μM)^1^	IC_50_ (μM)^2^	SI^3^
Paraiba/2015	ATA	Post	36	Vero	>1,000	13.87 ± 1.09	>72.10
Paraiba/2015	ATA	Post	36	A549	>1,000	33.33 ± 1.13	>26.26
Paraiba/2015	ARB	Post	36	Vero	74.71 ± 1.09	18.19 ± 1.16	4.11
Paraiba/2015	ARB	Post	36	A549	114.6 ± 1.08	51.87 ± 1.08	2.21
Uganda/1947	ATA	Post	48	Vero	>1,000	15.07 ± 1.19	>66.35
Nigeria/1968	ATA	Post	60	Vero	>1,000	15.97 ± 1.02	>62.62
Puerto Rico/2015	ATA	Post	48	Vero	>1,000	17.55 ± 1.16	>56.98
French Polynesia/2013	ATA	Post	48	Vero	>1,000	13.92 ± 1.01	>76.80
Brazil/2015	ATA	Pre	12	Vero	>1,000	14.33 ± 1.06	>69.78
Brazil/2015	ATA	Pre	24	Vero	>1,000	13.18 ± 1.05	>75.87
Brazil/2015	ATA	Pre	48	Vero	>1,000	12.59 ± 1.02	>79.42
Brazil/2015	ATA	Pre	72	Vero	>1,000	10.50 ± 1.05	>95.24


*^1^Median cytotoxicity concentration 50 (CC_50_). ^2^Median inhibitory concentration 50 (IC_50_). ^3^SI (Selective index) = CC_50_/IC_50_. For ATA, the SI was calculated considering a CC_50_ of 1,000 μM*.

### Inhibitory Effect of ATA on ZIKV Replication

**FIGURE 3:**
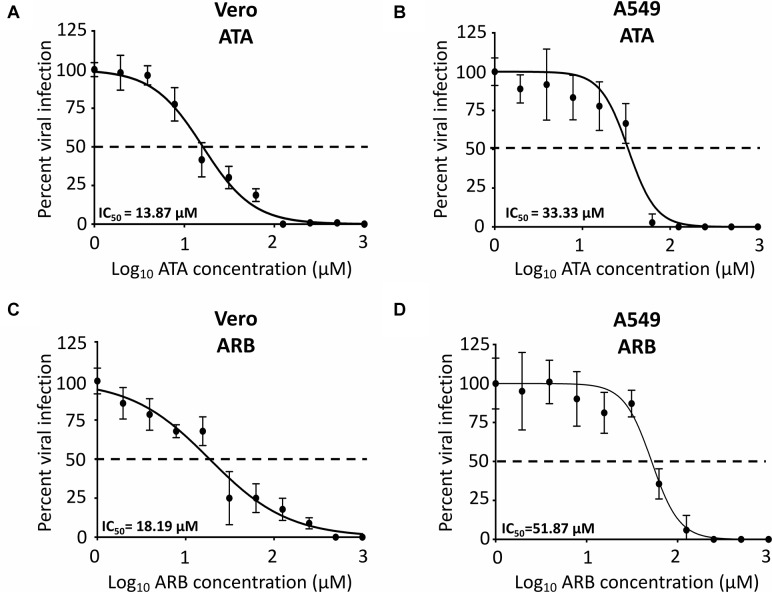
Inhibition of ZIKV Paraiba/2015 infection by ATA: Vero **(A,C)** or A549 **(B,D)** cells (96-well plate format, 5 × 10^4^ cells/well, triplicates) were infected with 25 PFU of Paraiba/2015. After 1 h of viral adsorption, the indicated concentrations (twofolds dilutions, starting concentration 1,000 μM) of ATA **(A,B)** or ARB **(C,D)** were added to 100 μl of infection media containing 1% Avicel. At 36 h p.i., infected cells were fixed for virus titration by immunostaining assay. Dotted line indicates 50% inhibition. Data was expressed as mean and SD from three independent experiments conducted in triplicates.

To determine the IC_50_ of ATA, Vero and A549 cells were infected with 25 PFU/well of Paraiba/2015 and after 1 h of viral absorption, virus inoculum was replaced with infection media with twofold serial dilutions (starting concentration of 1,000 μM) of ATA or ARB (Figure 3) and the IC_50_ calculated as described in the Section “Materials and Methods.” Although the IC_50_ of ATA (Figure 3A) and ARB (Figure 3C) in Vero cells were similar (13.87 ± 1.09 μM and 18.19 ± 1.6 μM, respectively), the selective index (SI, CC_50_/IC_50_) of ATA (>72.10) was significantly higher than that of ARB (4.11) (Table 1). Likewise, the IC_50_ of ATA (Figure 3B) and ARB (Figure 3D) in A549 cells were similar but with clearly different SI values (>26.26 for ATA and 2.21 for ARB) (Table 1). Notably the CC_50_, IC_50_, and SI of ARB were similar to those previously described in the literature in these cell lines (Fink et al., 2018; Haviernik et al., 2018). These data suggest that ATA exhibited an effective inhibition of ZIKV infection with limited toxicity and SI values better than those previously described for ARB.

**FIGURE 4:**
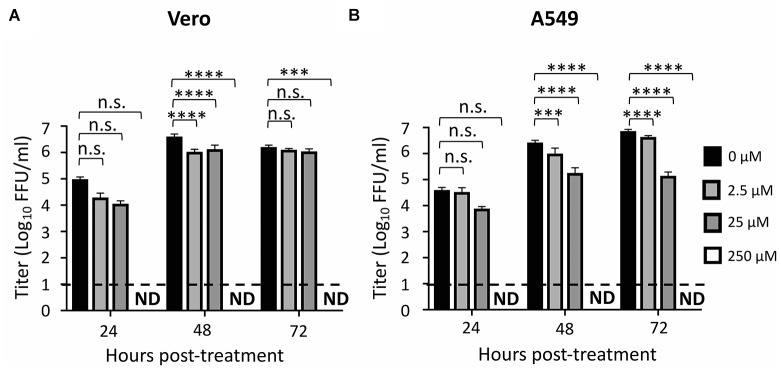
Aurintricarboxylic acid inhibition of ZIKV replication: Vero **(A)** and A549 **(B)** cells (24-well plate format, 2.5 × 10^5^ cells/well, triplicates) were infected (MOI 0.1) with Paraiba/2015. Tissue culture supernatants were collected at 24, 48, and 72 h p.i., and viral titer were calculated by immunostaining (fluorescent forming units, FFU/ml). Dotted line indicates the limit of detection (20 FFU/ml). Data was expressed as mean and standard deviations (SD) from three independent experiments conducted in triplicates. Statistical analysis was conducted by two-way ANOVA, ^∗^*p* < 0.0332, ^∗∗^*p* < 0.0021, ^∗∗∗^*p* < 0.0002, ^∗∗∗∗^*p* < 0.0001, or no significance (n.s.).

We also observed that ZIKV replication was completely inhibited at a concentration of 250 μM of ATA in Vero (Figure 4A) and A549 (Figure 4B) cells while 2.5 μM and 25 μM concentrations of ATA showed partial viral inhibition in Vero and A549 cells (Figure 4A,B), respectively, demonstrating a dose-dependent inhibition of viral replication in both cell lines.

### ATA Protects Cells From ZIKV-Induced Cell Death

**FIGURE 5:**
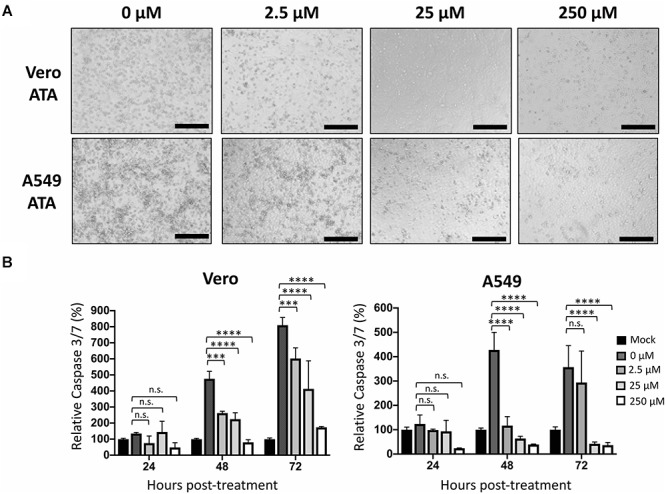
Aurintricarboxylic acid protects Vero and A549 cells from ZIKV-induced cell death: Vero and A549 cells (24-well plate format, 2.5 × 10^5^ cells/well, triplicates) were infected (MOI 0.1) with Paraiba/2015. After 1 h viral adsorption, cells were treated with the indicated concentrations (250, 25, 2.5, and 0 μM) of ATA. At 48 h p.i., cells were observed and imaged under an optical microscope. Scale bar = 100 μm. **(A)** Caspase 3/7 levels were measured in the tissue culture supernatants at 24, 48, and 72 h p.i. **(B)** Data of each time point was compared to mock-infected control cells and expressed as mean of relative percentage and SD from three independent experiments conducted in triplicates. Statistical analyses were conducted by two-way ANOVA, ^∗^*p* < 0.0332, ^∗∗^*p* < 0.0021, ^∗∗∗^*p* < 0.0002, ^∗∗∗∗^*p* < 0.0001, or no significance (n.s.).

We next evaluated the ability of ATA to protect cells from the cytopathic effect (CPE) induced during ZIKV infection (Figure 5). To that end, Vero and A549 cells were infected (MOI 0.1) with Paraiba/2015 and, after 1 h of viral absorption, cells were treated with 0, 2.5, 25, and 250 μM of ATA. At 48 h p.i., cells were observed under a light microscope for evaluation of their morphology and CPE (Figure 5A). As expected from our previous results, 25 μM and more clearly 250 μM of ATA were able to prevent ZIKV-induced CPE in both cell lines (Figure 5A). To quantify the ability of ATA to prevent ZIKV-induced apoptosis, tissue culture supernatants from ZIKV-infected Vero and A549 cells were harvested at 24, 48, and 72 h p.i. to measure the level of apoptotic signal as determined by caspase 3 and 7 activities (Figure 5B). ZIKV-infected cells showed increased caspase 3 and 7 levels up to eightfolds in Vero cells and up to 4.2-folds in A549 cells compared to mock-infected cells (Figure 5B). Levels of caspase 3 and 7 activation were dose-dependently reduced by ATA with 250 μM of ATA showed only 0.4- and 1.7-fold induction as compared to mock-infected Vero and A549 cells, respectively (Figure 5B).

### ATA Inhibition of Representative African and Asia/American ZIKV Strains

**FIGURE 6:**
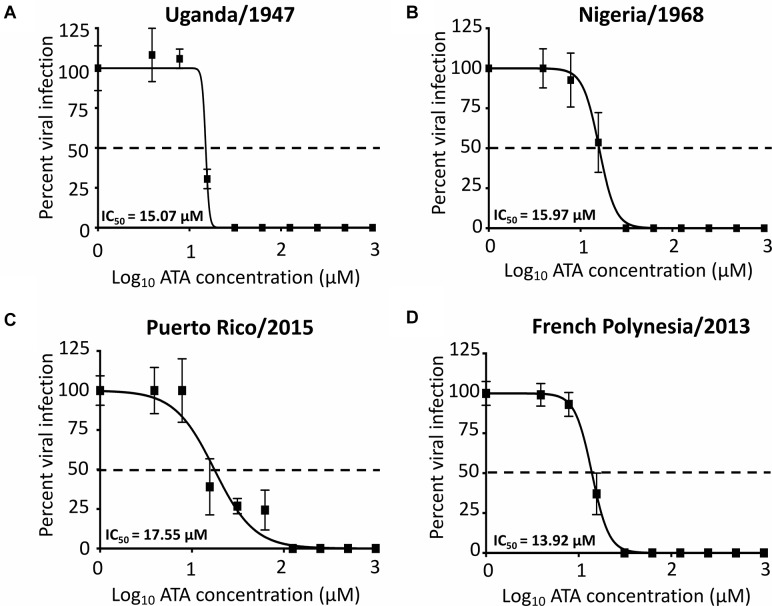
Aurintricarboxylic acid inhibition of African and Asian/American ZIKV strains: Vero cells (96-well plates, 5 × 10^4^ cells/well, triplicates) were infected with 25 PFU of Uganda/1947 **(A)**, Nigeria/1968 **(B)**, Puerto Rico/2015 **(C)**, and French Polynesia/2013 **(D)** ZIKV strains. After 1 h of viral adsorption, the indicated concentrations (twofolds dilutions, starting concentration 1,000 μM) of ATA were added to 100 μl of infection media containing 1% of Avicel. Infected cells were fixed for virus titration by immunostaining assay at 36–60 h p.i., depending on the ZIKV strains. Dotted line indicates 50% inhibition. Data was expressed as mean and SD from three independent experiments conducted in triplicates.

We next determined whether ATA is able to inhibit both ancestor African (Uganda/1947 and Nigeria/1968) and contemporary Asian/American (Puerto Rico/2015 and French Polynesia/2013) ZIKV lineage strains using our microplaque reduction assay (Figure 6). We observed similar IC_50_ values of ATA with Uganda/1947 (Figure 6A, IC_50_ = 15.07 ± 1.19 μM), Nigeria/1968 (Figure 6B, IC_50_ = 15.97 ± 1.02 μM), Puerto Rico/2015 (Figure 6C, IC_50_ = 17.55 ± 1.16 μM), and French Polynesia/2013 (Figure 6D, IC_50_ = 13.92 ± 1.01 μM), compared to those observed with Paraiba/2015 (Figure 3 and Table 1), demonstrating the broad antiviral activity of ATA against different ZIKV strains, regardless of the year and place of isolation.

### Pre-treatment of ATA Inhibits ZIKV Replication

**FIGURE 7:**
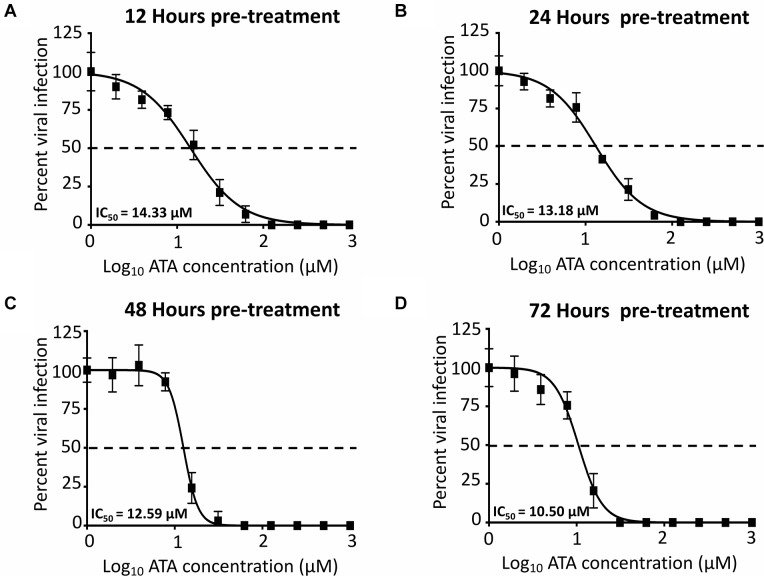
Inhibition of ZIKV by pre-treatment with ATA: Vero cells (96-well plates, 5 × 10^4^ cells/well, triplicates) were pre-treated with the indicated twofold dilution concentrations of ATA (starting concentration 1,000 μM) for 12 **(A)**, 24 **(B)**, 48 **(C)**, and 72 **(D)** h before infection (25 PFU) with ZIKV Paraiba/2015. At 36 h p.i., infected cells were fixed for virus titration by immunostaining. Dotted line indicates 50% inhibition. Data was expressed as mean and SD from three independent experiments conducted in triplicates.

To demonstrate the feasibility of using ATA for the prevention of ZIKV infection, important for travelers to regions where ZIKV is endemic, we next evaluated whether pre-treatment with ATA results in inhibition of ZIKV replication (Figure 7). To that end, Vero cells were pre-treated with ATA for 12 (Figure 7A), 24 (Figure 7B), 48 (Figure 7C), or 72 (Figure 7D) h prior to infection (MOI 0.1) with Paraiba/2015. Pre-treatment with ATA for 12–72 h before ZIKV infection resulted in similar IC_50_ values (14.33 ± 1.06 μM, 13.18 ± 1.05 μM, 12.59 ± 1.02 μM, and 10.50 ± 1.05 μM; respectively) demonstrating that ATA is stable and able to prevent ZIKV infection even when administered 3 days previous to viral infection (Figure 7 and Table 1).

## Discussion

The recent outbreak of ZIKV accompanied with severe pathology, including microcephaly in newborns, prompted many researchers to develop prophylactic vaccines and to identify therapeutic drugs against ZIKV infection (Abbink et al., 2016; Larocca et al., 2016; Lessler et al., 2016; Shan et al., 2017; Fink et al., 2018). Currently, there are no commercially available vaccines and/or antiviral therapies for the treatment of ZIKV infection. Therefore, there is an urgent medical need for the development of effective counter measurements to control ZIKV infection.

In this study, we demonstrated that ATA (Figure 1) has limited toxicity (Figure 2) and an effective and dose-dependent antiviral activity against ZIKV infection (Figure 3, 4) in both monkey kidney epithelial Vero and human alveolar A549 cells. Notably, ATA can prevent ZIKV-induced CPE and apoptosis in both cell lines (Figure 5) and has broad anti-viral activity against representative ZIKV strains from the African (Uganda/1947 and Nigeria/1968) and the Asian/American (Puerto Rico/2015 and French Polynesia/2013) lineages (Figure 6). Moreover, ATA can also prevent ZIKV infection even when administered 3 days before infection (Figure 7).

Aurintricarboxylic acid is a polyanionic aromatic compound that structurally relates to suramin (Balzarini et al., 1986) and is believed to influence over 100 host and viral enzymes (Shadrick et al., 2013). Although the exact mechanism by which ATA inhibits ZIKV infection was not identified in this study, there are several plausible mechanisms on ZIKV inhibition mediated by ATA, including the targeting of viral and cellular proteins. In terms of inhibiting viral proteins, ATA could bind to ZIKV NS3 helicase and prevent its binding to either ATP or nucleic acids, as previously described for HCV (Mukherjee et al., 2012; Shadrick et al., 2013). Likewise, ATA could inhibit the ZIKV RNA-dependent RNA polymerase (RdRp) NS5 protein, as described for HCV (Chen et al., 2009; Mukherjee et al., 2012; Shadrick et al., 2013) and enterovirus 71 (Hung et al., 2010). Similarly, ATA could inhibit the methyltransferase activity of NS5 involved in mRNA capping processes, as previously described for other Flaviviruses (DENV and YFV) (Milani et al., 2009; Garcia et al., 2017). Because of the structural similarities between DENV and ZIKV NS5 proteins, it is feasible that, similar to DENV, ATA binds to NS5 to inhibit ZIKV infection (Garcia et al., 2017). Moreover, it is possible that ATA targets and has inhibitory activities against one or more of the viral proteins described above.

In terms of targeting cellular proteins important for the efficient replication of ZIKV, it has been previously described that ATA has anti-apoptotic properties in a variety of cells (Chen et al., 2002). It is possible that the anti-apoptotic activity of ATA protects against ZIKV-induced cell death, as demonstrated in this study (Figure 5). Notably, it has been recently shown that ZIKV infection induced apoptosis through caspase 3 and 9 in A549 cells and through caspase 3 in neonatal mice brain (Huang et al., 2016; Frumence et al., 2016). These results suggest that inhibition of ZIKV replication results in a decrease in the level of apoptotic cells and that the anti-apoptotic effect of ATA affects ZIKV replication. Further research is guaranteed to yield a better understanding of the antiviral activity of ATA on ZIKV infection, and other viruses, before the use of ATA as an antiviral drug.

During January 2015 to February 2016, a total of 116 residents from 33 states in the United States were diagnosed with ZIKV infection (Armstrong et al., 2016). Out of 115 patients, 110 (96%) traveled to areas of active ZIKV transmission before the infection and five (4%) did not travel but reported sexual contact with a traveler who had a symptomatic illness (Armstrong et al., 2016). For these reasons, preventive efforts are required prior to travel to areas of active ZIKV transmission. In this study, cells pretreated with ATA for up to 72 h prior to infection with ZIKV showed similar IC_50_ than those in post-treatment settings, potentially suggesting that ATA might target a cellular protein required for ZIKV replication or that the concentration and stability of ATA in pre-treated cells is sufficient to inhibit ZIKV infection, or both. Nevertheless, these results demonstrate the feasibility of using ATA for the prophylactic treatment of viral infection, including those traveling to areas where ZIKV is endemic. Moreover, due the broad inhibition effect of ATA against others viruses and parasites (Liang et al., 2003; He et al., 2004; De Clercq, 2005; Myskiw et al., 2007; Klein et al., 2008; Chen et al., 2009; Hung et al., 2009, 2010; Mukherjee et al., 2012; Shadrick et al., 2013) that are present in ZIKV endemic areas, treatment with ATA could be used for the broad prevention of DENV, YFV (Milani et al., 2009; Shadrick et al., 2013; Garcia et al., 2017), HCV (Chen et al., 2009; Mukherjee et al., 2012; Shadrick et al., 2013), and parasitic infestation (*Cryptosporidium parvum*) (Klein et al., 2008) for people traveling to these endemic regions. Moreover, the broad spectrum antiviral activity of ATA against different African and Asian/American ZIKV strains further guarantees the feasibility of implementing ATA to prevent ZIKV infection to travelers around the world.

Although ATA has been amply evaluated *in vitro*, only few studies have assessed the activity of ATA *in vivo*, including its use as a curative agent against thrombosis (Strony et al., 1990), apoptosis (Roberts-Lewis et al., 1993; Heiduschka and Thanos, 2000), parasite infestations (Klein et al., 2008), bacterial (*Y. pestis*) (Liang et al., 2003), and Vaccinia virus (Smee et al., 2010) infections. In the case of Vaccinia virus, ATA did not protect mice from a lethal challenge at a dose of 30 mg/kg/day (Smee et al., 2010). Further studies are needed to evaluate the anti-viral activity of ATA *in vivo* for the treatment of viral infections, including ZIKV.

Our studies show limited toxicity, if any, of ATA in cultured cells, including human A549 cells. The lack of knowledge about the use of ATA in pregnant women requires future additional safety tests, including studies using validated animal models of ZIKV infection, before using ATA for the treatment of ZIKV infection during pregnancy. Based on the effectiveness of ATA against ZIKV infection (SI = 72.1 in Vero cells and 26.26 in A549 cells) as compared to other previously described drugs, including emetine (SI = 9.84 in SNB-19 cells and 2.88 in ENV+ cells) (Yang et al., 2018), obatoclax [SI = 65 in human retinal pigment epithelial (RPE) cells] (Kuivanen et al., 2017), saliphenylhalamide (SI > 200 in RPE cells) (Kuivanen et al., 2017), gemcitabine (SI > 1,000 in RPE cells) (Kuivanen et al., 2017), sofosbuvir (SI > 52.63 in Huh-7 cells and 90.9 in Jar cells) (Bullard-Feibelman et al., 2017) and ARB (SI = 4.11 in Vero cells and 2.21 in A549 cells) (Fink et al., 2018; Haviernik et al., 2018) and this study (ATA, SI = 56.98–95.24 in Vero cells, and 26.26 in A549 cells), it is possible that ATA represents one of the most reasonable options of the treatment of ZIKV infection.

## Author Contributions

J-GP, GÁ-P, and FM conceived, designed, and performed the experiments. TH, AN, FA, and LM-S provided the reagents, suggestions, and resources. FA and LM-S provided the funds. J-GP analyzed the data and wrote the initial manuscript draft. GÁ-P, FM, TH, AN, FA, and LM-S edited the original draft.

## Conflict of Interest Statement

The authors declare that the research was conducted in the absence of any commercial or financial relationships that could be construed as a potential conflict of interest.
